# One Year Later: What Was the Impact of the COVID-19 Pandemic on Orthopedic Practice?

**DOI:** 10.7759/cureus.16013

**Published:** 2021-06-29

**Authors:** Elias Vasiliadis, Christos Vlachos, Eftychios Papagrigorakis, Dimitrios Stergios Evangelopoulos, Moyssis Lelekis, Spyros G Pneumaticos

**Affiliations:** 1 3rd Orthopedic Department, University of Athens, KAT General Hospital of Athens, Athens, GRC; 2 3rd Orthopedic Department, School of Medicine, National and Kapodistrian University of Athens, KAT General Hospital of Athens, Athens, GRC; 3 3rd Orthopedics Department, School of Medicine, National and Kapodistrian University of Athens, KAT General Hospital of Athens, Athens, GRC

**Keywords:** covid-19, pandemic, trauma, elective, surgeries, orthopedic

## Abstract

The coronavirus disease 2019 (COVID-19) pandemic is an enormous challenge for health care systems worldwide.

Although it is widely accepted that orthopedic service has been reduced during the COVID-19 pandemic, little is known about the magnitude and qualitative characteristics of this reduction. The aim of the present study is to quantify the impact of the COVID-19 pandemic on everyday orthopedic practice and to detect the qualitative details of this impact in order to provide data for appropriate planning of health care policy.

Data from the year 2020, when the COVID-19 pandemic occurred, regarding the number of patients examined in the emergency department, outpatient clinics, as well as the number of hospital admissions, were recorded for each month. The number of surgical procedures per month was also recorded and evaluated in relation to the category and the anatomical region that these procedures pertained to. Similar data from the year 2019 were used as a control group.

The mean number of patients who visited the emergency department, the outpatient clinics, and those who were admitted to the hospital per month decreased by 47.2%, 30.4%, and 9%, respectively. Overall, the mean number of orthopedic operations decreased by 11.7%, with trauma operations being reduced by 8.9% and elective operations by 13% per month.

Based on the findings of the present study, the impact of the COVID-19 pandemic on orthopedic patients is definitely negative. The establishment of new guidelines and re-distribution of resources is required to return to a normal function of orthopedic practice within hospitals.

## Introduction

The coronavirus disease 2019 (COVID-19) pandemic is an enormous challenge for health care systems worldwide. Medical practice has shifted toward the treatment of patients with the COVID-19 disease. Both human and economic resources are redistributed for the creation of additional facilities to meet the increased requirements of those patients.

Orthopedic practice has definitely been affected both for its elective and emergency service [1]. Patients with chronic musculoskeletal disorders are advised to postpone definitive treatment if possible and access to outpatient clinics is restricted following guidelines issued by medical authorities. On the other hand, emergency orthopedic service remains unaltered, and all trauma patients receive appropriate treatment as they did before the pandemic. Although it is widely accepted that orthopedic service has been reduced during the COVID-19 pandemic, little is known about the magnitude and qualitative characteristics of this reduction.

The first COVID-19 patient in Greece was diagnosed on February 26, 2020, and since then the pandemic has spread across the whole country. Health authorities issued restrictions for most social activities since then, and from March 23, 2020, a strict lockdown was applied in the whole country for six weeks. A gradual restart of social activities was allowed by the end of June 2020 although recommendations regarding social distancing and mask-wearing remained. Following a second wave of the pandemic, a new lockdown was ordered commencing November 9, 2020, until the end of the year. During the first lockdown, all hospital admissions were suspended except trauma and emergency patients. Outpatient clinics were canceled and elective operations were postponed. During the second lockdown, government directives were to diminish elective procedures and outpatient clinic attendances by 80% to overload the health care system and to preserve stuff and equipment for COVID-19 patients [2].

The aim of the present study is to quantify the impact of the COVID-19 pandemic in everyday orthopedic practice and to detect the qualitative details of this impact in order to provide data for appropriate planning of health care policy.

## Materials and methods

The study was performed in an academic orthopedic department within a public hospital in Greece, which is a tertiary referral center for both elective and emergency practice. After ethical approval by the Institutional Review Board of KAT Hospital, medical records of all patients who were admitted in our department in 2019 and 2020 were retrospectively analyzed. Patient hospitalization, outpatient clinics, and emergency department visits, elective and trauma operations for each month of the above two-year period were recorded.

Our department is in a non-COVID-19 hospital, although there is an intensive care unit dedicated to treating COVID-19 patients. During the first six months of the pandemic, every new admission was screened for COVID-19 symptoms, and a thorough travel and contact history was recorded. For patients at risk, a reverse transcription-polymerase chain reaction (RT-PCR) test for severe acute respiratory syndrome coronavirus 2 (SARS-COV-2) was carried out. Since July 1, 2020, a negative RT-PCR test was required from all patients prior to admission to the hospital.

For each month of 2019 and 2020, differences in the number of patients examined in the emergency department and the outpatient clinics, as well as the number of patients admitted to our department for further evaluation, were recorded. The number of surgical procedures was also recorded and evaluated in relation to the category (emergency or elective) and the anatomical location that these procedures were performed.

Data from the year 2019 were used as a control group since the operation of our department had not yet been affected by the COVID-19 pandemic. Differences between each month of 2019 and 2020 were documented. Data were analyzed separately for each month because the impact of the COVID-19 pandemic was not equally distributed, as different restrictions and guidelines were in use throughout the year 2020.

Statistical analysis was performed using the IBM Statistical Package for the Social Sciences (SPSS) v. 22.0 for Windows (IBM Corp, Armonk, NY). Descriptives of all the examined variables are provided for each month of the examined years. Continuous cohort variables were compared using the Mann-Whitney U test with Bonferroni correction to adjust for multiple comparisons. P-value <.05 was considered statistically significant.

## Results

A mean decrease of 47,2% in the mean value was recorded when comparing the number of patients examined in the emergency department during the pre-pandemic year 2019 and the pandemic year 2020. The number of visits dropped from 1042.3±160.3 per month in 2019, to 550.3 ± 239.2 per month in 2020 (p < 0.001) (Table 1).

**Table 1:** Changes in the total number of patients examined in the emergency department and outpatient clinics, the admissions, and the number of trauma and elective operations performed in the years 2019 (before the COVID-19 pandemic) and 2020 (during the COVID-19 pandemic)

	2019		2020			
	Mean ± SD	Median (IQR)	Mean ±SD	Median (IQR)	Change	p-value
Emergency Department	1042.33 ± 160.310	1038.0 (716-1365)	550.33 ± 239.241	474 (246-996)	-47.20%	<0.001
Admissions	186.58 ± 21.878	191.50 (146-211)	169.83 ± 41.264	173 (89-223)	-8.98%	0.436
Outpatient Clinics	509 ± 70.253	517 (340-602)	354.17 ± 97.855	360 (166-473)	-30.42%	<0.001
Total Number of Operations	88.42 ± 10.808	91 (64-105)	78.08±14.094	80 (47-104)	-11.69%	0.035
Trauma Operations	58.17 ± 6.206	58 (46-68)	53 ± 11.481	48.50 (41-77)	-8.88%	0.088
Elective Operations	28.92 ± 8.295	29.50 (8-40)	25.17 ± 12.164	26.50 (1-40)	-12.97%	0.470

**Figure 1:**
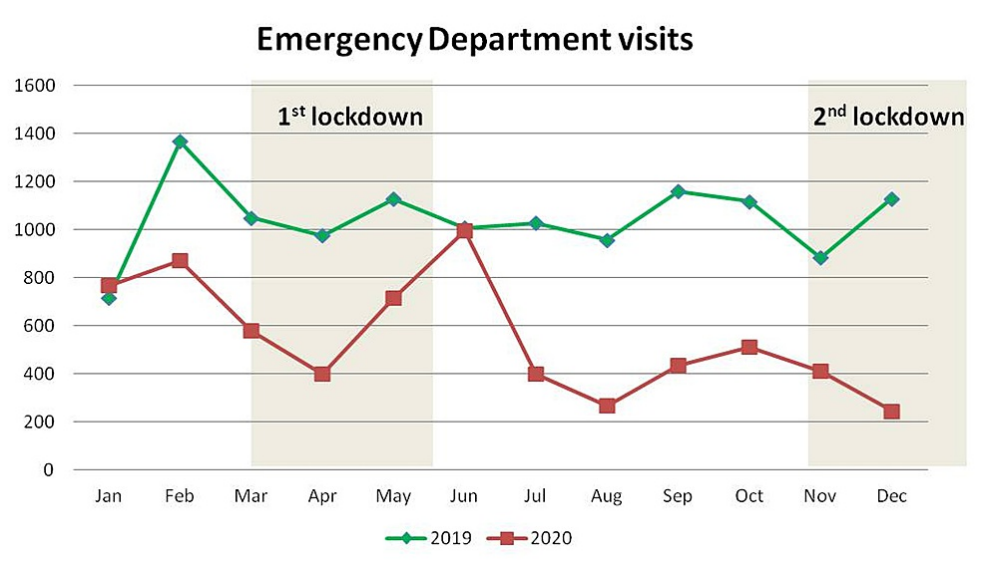
Monthly distribution of the number of patients who visited the emergency department

This drop was noted during the whole year of 2020, except January, when the COVID-19 pandemic was not present, and June, when restriction measures had been withdrawn (Table 2; Figure 1).

**Table 2:** Number of patients examined per month of 2019 and 2020 in the emergency department, and their change in percentage

Emergency Department			
Month	2019	2020	Change
Jan	716	768	7.3%
Feb	1365	872	-36.1%
Mar	1049	581	-44.6%
Apr	974	399	-59.0%
May	1126	715	-36.5%
Jun	1006	996	-1.0%
Jul	1027	400	-61.1%
Aug	957	267	-72.1%
Sep	1158	436	-62.3%
Oct	1117	512	-54.2%
Nov	884	412	-53.4%
Dec	1129	246	-78.2%

**Figure 2:**
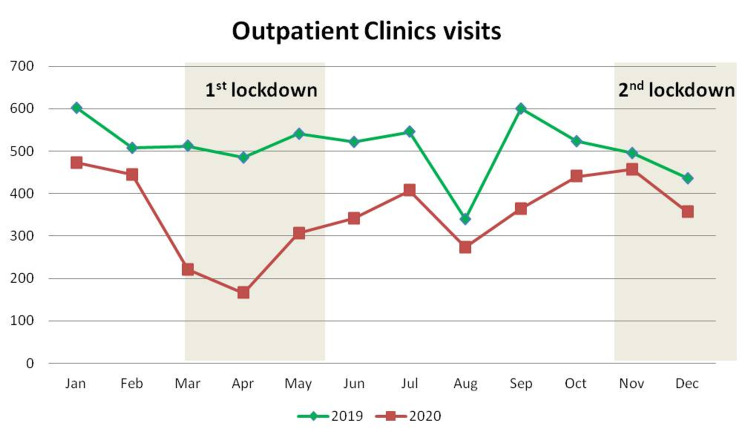
Monthly distribution of the number of patients examined at the outpatient clinics

The mean value of the total number of visits in the outpatient clinics fell by 30.4%, from 509 ± 70.3 per month in 2019 to 354.2 ± 97.86 per month in 2020 (p < 0.001) (Table 3). This reduction was documented during the entire 2020 but was more prominent during March, April, and May, when the first lockdown had been issued (Table 3; Figure 2).

**Table 3:** Number of patients examined per month of 2019 and 2020 in the outpatient clinics and their change in percentage

Outpatient Clinics			
Month	2019	2020	Change
Jan	602	473	-21.4%
Feb	507	445	-12.2%
Mar	512	221	-56.8%
Apr	485	166	-65.8%
May	541	307	-43.6%
Jun	522	341	-34.7%
Jul	545	407	-25.3%
Aug	340	273	-19.7%
Sep	600	364	-39.3%
Oct	523	440	-15.9%
Nov	495	457	-7.7%
Dec	436	356	-18.3%

**Figure 3:**
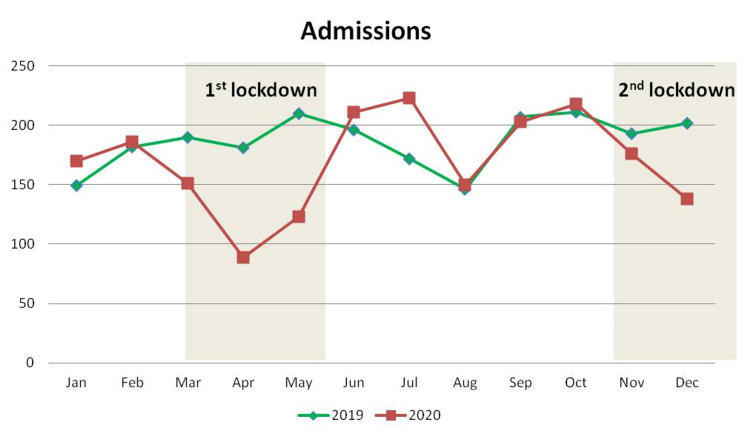
Monthly distribution of the number of patients admitted to the hospital

The mean value of hospital admissions decreased by 9% from 186.6 ± 21.9 per month in 2019 to 169.8 ± 41.3 per month in 2020. This difference was not statistically significant (Table 1). When studying hospital admissions by month, it was found that a noticeable reduction was present during March (20.5%), April (50.8%), and May (41.4%) of 2020 as well as December (31.7%) of 2020 when the first and second lockdown had been ordered respectively (Table 4; Figure 3).

**Table 4:** Numbers of hospital admissions per month of 2019 and 2020 and their change in percentage

Admissions			
Month	2019	2020	Change
Jan	149	170	14.1%
Feb	182	186	2.2%
Mar	190	151	-20.5%
Apr	181	89	-50.8%
May	210	123	-41.4%
Jun	196	211	7.7%
Jul	172	223	29.7%
Aug	146	150	2.7%
Sep	207	203	-1.9%
Oct	211	218	3.3%
Nov	193	176	-8.8%
Dec	202	138	-31.7%

**Figure 4:**
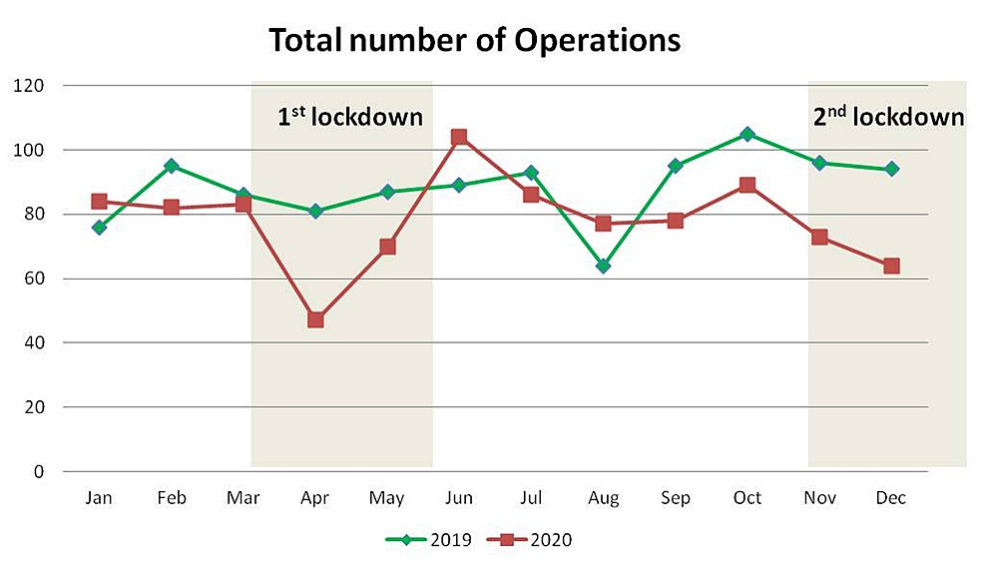
Monthly distribution of the total number of orthopedic operations

The mean value of overall orthopedic operations (elective as well as trauma) performed in our department decreased by 11.7% from 88.4 ± 10.8 per month of 2019 to 78.1 ± 14.1 per month of 2020 (p=0.035). This reduction was evident throughout 2020, except for January and August, when an increase of 20.3% was recorded (Table 5; Figure 4).

**Table 5:** Total number of orthopedic operations per month of 2019 and 2020 and their change in percentage

Number of operations			
Month	2019	2020	Change
Jan	76	84	10.5%
Feb	95	82	-13.7%
Mar	86	83	-3.5%
Apr	81	47	-42.0%
May	87	70	-19.5%
Jun	89	104	16.9%
Jul	93	86	-7.5%
Aug	64	77	20.3%
Sep	95	78	-17.9%
Oct	105	89	-15.2%
Nov	96	73	-24.0%
Dec	94	64	-31.9%

**Figure 5:**
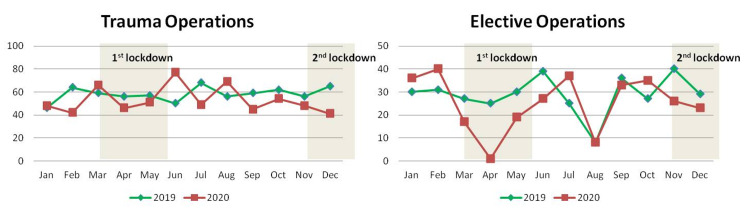
Monthly distribution of the number of trauma and elective operations

The mean value of trauma operations fell by 8.9% from 58.2 ± 6.2 per month of 2019 to 53 ± 11.5 per month of 2020 while elective operations fell by 13% from 28.9 ± 8.3 per month of 2019 to 25.2 ± 12.2 per month of 2020. The reduction in both trauma and elective operations was not statistically significant (Table 1). The difference in the number of operations was not equally distributed throughout 2020 and a considerable variation was manifested between months (Table 6; Figure 5).

**Table 6:** Numbers of trauma and elective operations per month of 2019 and 2020 and their change in percentage

Trauma Operations				Elective Operations		
Month	2019	2020	Change	2019	2020	Change
Jan	46	48	4.3%	30	36	20.0%
Feb	64	42	-34.4%	31	40	29.0%
Mar	59	66	11.9%	27	17	-37.0%
Apr	56	46	-17.9%	25	1	-96.0%
May	57	51	-10.5%	30	19	-36.7%
Jun	50	77	54.0%	39	27	-30.8%
Jul	68	49	-27.9%	25	37	48.0%
Aug	56	69	23.2%	8	8	0%
Sep	59	45	-23.7%	36	33	-8.3%
Oct	62	54	-12.9%	27	35	29.6%
Nov	56	48	-14.3%	40	26	-35.0%
Dec	65	41	-36.9%	29	23	-20.7%

Analysis of trauma operations revealed a reduction in hip procedures by 19.6% from 25.5 ± 7.2 per month of 2019 to 20.5 ± 4.0 per month of 2020. Lower limb procedures were reduced by 2.6% from 15.8 ± 2.6 per month of 2019 to 15.4 ± 4.6 per month of 2020. Spinal procedures were reduced by 34.8% from 1.9 ± 1.4 per month of 2019 to 1.3 ± 1.3 per month of 2020 and infections were reduced by 2.9% from 8.6 ± 3.4 per month of 2019 to 8.3 ± 4.8 per month of 2020. Upper limb procedures showed an increase of 24.6% from 5.4 ± 2.9 per month of 2019 to 6.8 ± 3.4 per month of 2020. None of the above changes was statistically significant after Bonferroni correction for multiple testing. Details and absolute numbers are shown in Table 7.

**Table 7:** Changes in the number of trauma and elective operations performed in the years 2019 (before the COVID-19 pandemic) and 2020 (during the COVID-19 pandemic)

	2019		2020			
	Mean ± SD	Median (IQR)	Mean ±SD	Median (IQR)	Change	p value
Trauma Hip Operations	25.5 ± 7.154	26 (15-39)	20.5 ± 3.966	22.5 (11-25)	-19.61%	0.059
Trauma Lower Limb Operations	15.83 ± 2.552	16 (11-20)	15.42 ± 4.582	13.5 (10-24)	-2.63%	0.451
Trauma Upper Limb Operations	5.42 ± 2.948	5.5 (1-11)	6.75 ± 3.441	7 (2-15)	+24.62%	0.307
Trauma Spinal Operations	1.92 ± 1.442	2 (0-4)	1.25 ± 1.288	1 (0-4)	-34.78%	0.236
Infections	8.58 ± 3.370	8.5 (2-15)	8.33 ± 4.774	8 (1-20)	-2.9%	0.706
Total Hip Arthroplasty	8.08 ± 4.188	8 (2-17)	6.33 ± 4.271	7.5 (0-12)	-21.65%	0.562
Total Knee Arthroplasty	3.83 ± 1.801	4 (2-8)	3.08 ± 2.353	3.5 (0-6)	-19.57%	0.493
Elective Shoulder Operations	1.25 ± 0.662	1 (0-2)	1.33 ± 1.371	1 (0-4)	+6.67%	0.713
Elective Spinal Operations	6.42 ± 2.968	6 (0-12)	4.42 ± 2.193	5 (0-8)	-31.17%	0.048
Deformities	4 ± 2.558	3.5 (1-8)	3.92 ± 2.999	3 (1-10)	-2.08%	0.861
Knee Arthroscopy	6.67 ± 3.143	6 (3-12)	6.08 ± 4.337	5.5 (0-14)	-8.75%	0.542

**Figure 6:**
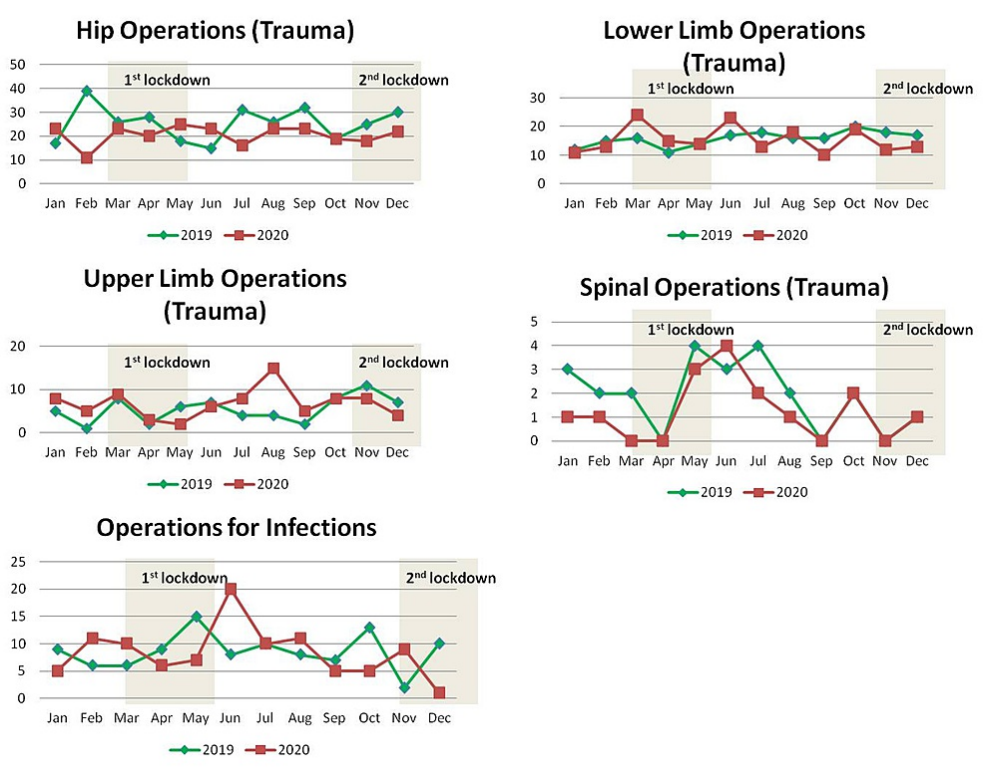
Monthly distribution of the number of trauma operations by category

Details for the different categories of trauma operations and their monthly distribution for the years 2019 and 2020, respectively, are shown in Table 8 and Figure 6.

**Table 8:** Monthly distribution of different categories of trauma operations in the years 2019 (before the COVID-19 pandemic) and 2020 (during the COVID-19 pandemic)

	Hip Operations			Lower Limb Operations			Upper Limb Operations			Spinal Operations			Infections		
Month	2019	2020	%	2019	2020	%	2019	2020	%	2019	2020	%	2019	2020	%
Jan	17	23	35.3%	12	11	-8.3%	5	8	60.0%	3	1	-66.7%	9	5	-44.4%
Feb	39	11	-71.8%	15	13	-13.3%	1	5	400.0%	2	1	-50.0%	6	11	83.3%
Mar	26	23	-11.5%	16	24	50.0%	8	9	12.5%	2	0	-100.0%	6	10	66.7%
Apr	28	20	-28.6%	11	15	36.4%	2	3	50.0%	0	0	0%	9	6	-33.3%
May	18	25	38.9%	14	14	0%	6	2	-66.7%	4	3	-25.0%	15	7	-53.3%
Jun	15	23	53.3%	17	23	35.3%	7	6	-14.3%	3	4	33.3%	8	20	150.0%
Jul	31	16	-48.4%	18	13	-27.8%	4	8	100.0%	4	2	-50.0%	10	10	0%
Aug	26	23	-11.5%	16	18	12.5%	4	15	275.0%	2	1	-50.0%	8	11	37.5%
Sep	32	23	-28.1%	16	10	-37.5%	2	5	150.0%	0	0	0%	7	5	-28.6%
Oct	19	19	0%	20	19	-5.0%	8	8	0%	2	2	0%	13	5	-61.5%
Nov	25	18	-28.0%	18	12	-33.3%	11	8	-27.3%	0	0	0%	2	9	350.0%
Dec	30	22	-26.7%	17	13	-23.5%	7	4	-42.9%	1	1	0%	10	1	-90.0%

**Figure 7:**
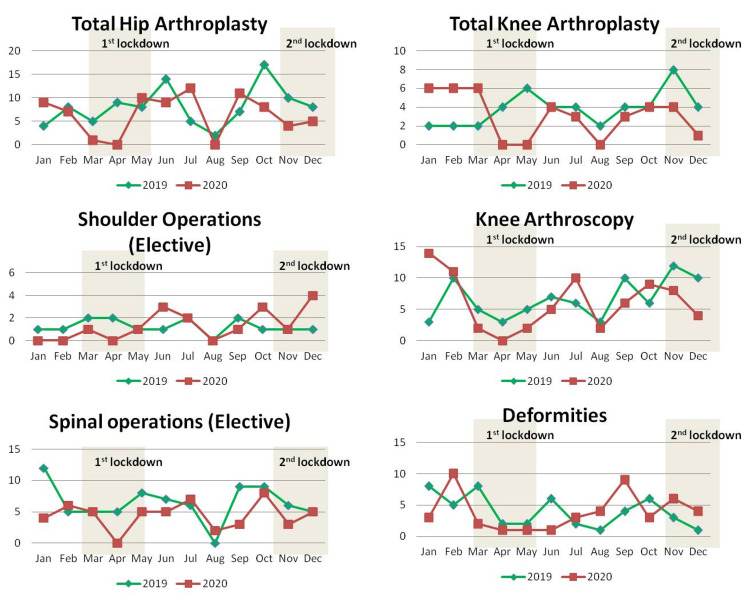
Monthly distribution of the number of elective operations by category

Analysis of elective operations showed a decrease of total hip arthroplasties by 21.7% from 8.1 ± 4.2 per month of 2019 to 6.3 ± 4.3 per month of 2020. Total knee arthroplasties were decreased by 19.6% from 3.8 ± 1.8 per month of 2019 to 3.1 ± 2.4 per month of 2020. Spinal operations were decreased by 31.2% from 6.4 ± 3.0 per month of 2019 to 4.4 ± 2.2 per month of 2020. Knee arthroscopies were decreased by 8.8% from 6.7 ± 3.143 per month of 2019 to 6.1 ± 4.3 per month of 2020 and deformity corrections were decreased by 2.1% from 4 ± 2.6 per month of 2019 to 3.9 ± 3.0 per month of 2020. Shoulder operations were increased by 6.7% from 1.3 ± 0.7 per month of 2019 to 1.3 ± 1.4 per month of 2020. After Bonferroni correction for multiple testing, all changes, except for the reduction of elective spinal operations, were not statistically significant (Table 7). Monthly distribution and absolute numbers of different categories of elective operations are shown in detail in Table 9 and Figure 7.

**Table 9:** Monthly distribution of different categories of elective operations in the years 2019 (before the COVID-19 pandemic) and 2020 (during the COVID-19 pandemic)

Total Hip Arthroplasty				Total Knee Arthroplasty			Shoulder Operations			Spinal Operations			Deformities			Knee Arthroscopy		
Month	2019	2020	%	2019	2020	%	2019	2020	%	2019	2020	%	2019	2020	%	2019	2020	%
Jan	4	9	125.0%	2	6	200.0%	1	0	-100.0%	12	4	-66.7%	8	3	-62.5%	3	14	366.7%
Feb	8	7	-12.5%	2	6	200.0%	1	0	-100.0%	5	6	20.0%	5	10	100.0%	10	11	10.0%
Mar	5	1	-80.0%	2	6	200.0%	2	1	-50.0%	5	5	0%	8	2	-75.0%	5	2	-60.0%
Apr	9	0	-100.0%	4	0	-100.0%	2	0	-100.0%	5	0	-100.0%	2	1	-50.0%	3	0	-100.0%
May	8	10	25.0%	6	0	-100.0%	1	1	0%	8	5	-37.5%	2	1	-50.0%	5	2	-60.0%
Jun	14	9	-35.7%	4	4	0%	1	3	200.0%	7	5	-28.6%	6	1	-83.3%	7	5	-28.6%
Jul	5	12	140.0%	4	3	-25.0%	2	2	0%	6	7	16.7%	2	3	50.0%	6	10	66.7%
Aug	2	0	-100.0%	2	0	-100.0%	0	0	0%	0	2	0%	1	4	300.0%	3	2	-33.3%
Sep	7	11	57.1%	4	3	-25.0%	2	1	-50.0%	9	3	-66.7%	4	9	125.0%	10	6	-40.0%
Oct	17	8	-52.9%	4	4	0%	1	3	200.0%	9	8	-11.1%	6	3	-50.0%	6	9	50.0%
Nov	10	4	-60.0%	8	4	-50.0%	1	1	0%	6	3	-50.0%	3	6	100.0%	12	8	-33.3%
Dec	8	5	-37.5%	4	1	-75.0%	1	4	300.0%	5	5	0%	1	4	300.0%	10	4	-60.0%

## Discussion

The government, in an effort to control the COVID-19 pandemic, imitated other countries’ measures and adopted a strict lockdown during the two peaks of the COVID-19 pandemic, with a small quarantine-free period during summer months. People were instructed to stay home in order to stay safe, transportation between districts was prohibited, remote working was established, schools and universities suspended their operation, retail and leisure places were closed, and social distancing and mask-wearing became compulsory in an effort to eliminate the transmission of the virus among the population. As a consequence, people limited their activities and restrained visits to medical centers for chronic conditions mainly because they fear COVID-19 contamination. A notable decrease in patient numbers seeking medical consultation was monitored in hospitals, including orthopedic patients [1,3].

Initially, a significant decrease was reported in the number of patients who visited the emergency department in 2020 as compared to 2019. This is attributed to the restriction of most outdoor activities, leading to less traffic, occupational accidents, and sports injuries. Additionally, many patients with minor injuries did not ask for medical support, due to the fear of coming in contact with the COVID-19 virus or even due to altruistic motivation for health care workers [4].

Similarly, a significant reduction was found in the number of patients examined in the outpatient clinics of our department throughout the whole year of 2020. This reduction was in accordance with the hospital’s administrative policy, which reduced the available appointments and increased the waiting time between patients in order to avoid overcrowding and protect health care workers from virus contamination [5-6].

On the contrary, hospital admissions significantly decreased during the first lockdown in March, April, and May 2020, but this tendency was not so evident during the second lockdown in November and December 2020. Furthermore, a minor increase in hospital admissions was noticed during the summer months. Loosening of restrictions during summer for a quarantine-free tourist period and lighter restrictions during the second lockdown are possible explanations for these findings. Consequently, these strategies led to a rise in the number of trauma patients and those looking for the treatment of chronic orthopedic disease.

Compared with the significant reduction in the visits to the emergency department, the reduction of emergency operations was disproportionally lower, mainly because the hospital is a tertiary referral center and patients who visited our department were referred for operative management rather than simple orthopedic consultation. Hip fractures presented a rather stable monthly distribution throughout 2020 and when compared to 2019, their decrease was not statistically significant, as they represent a fragility type of fracture, occur after in-house falls, and mainly affect elderly people with many co-morbidities [7]. Lower limb trauma operations did not present any significant change. Interestingly, upper limb trauma operations increased, possibly because they are related to indoor activities, which were increased during the quarantine period. Emergency spinal operations were decreased mainly because road accidents and falls from height were diminished. The number of orthopedic infections that needed surgical treatment was similar between 2019 and 2020. Periprosthetic joint infections, osteomyelitis, and septic arthritis are considered surgical emergencies and their number remained unaffected, as they represent complications of previous surgical interventions in the pre-pandemic period.

Restriction of elective operations was among the first measures that were adopted after the outbreak of the COVID-19 pandemic. Health authorities postponed the majority of elective surgeries during the first lockdown and reduced their number by 80% during the second lockdown in an effort to preserve recourses and equipment for the treatment of COVID-19 patients [8]. Despite this policy, the reduction of elective operations during 2020 in our department was less marked. Apart from elective spinal operations, which presented a statistically significant decrease, the number of joint reconstruction procedures, knee arthroscopy, shoulder elective operations, and deformity corrections showed a lower decrease and followed a biphasic monthly distribution with a significant reduction during the lockdown months and a counterbalance during the rest of 2020 as compared to 2019. One possible explanation is that many elective orthopedic operations are considered as “urgent-elective” due to the severe disability they imply for the patient and are therefore prioritized as soon as restrictive measures are alleviated [9-10].

It is still unclear whether elective surgeries will return to their normal flow; however, it is certain that once this happens, the health care system will be confronted with a huge backlog of elective surgeries [11].

## Conclusions

Based on the findings of the present study, the impact of the COVID-19 pandemic on orthopedic practice is definitely negative. The access of patients with orthopedic problems is restricted and elective operations are postponed following restrictions issued by policy-makers. It is not clear what the consequences of the currently applied policy will be in the long term. It is crucial to re-establish the guidelines in order to reschedule the elective operations. There is no doubt that precautions for virus spread must be enforced and supplementary resources should be re-distributed in order to return to the normal function of orthopedic practice; otherwise, the negative impact of the COVID-19 pandemic will be sustained and many disabled patients with orthopedic diseases will lose any access to treatment.
